# VO_2_-based ultra-reconfigurable intelligent reflective surface for 5G applications

**DOI:** 10.1038/s41598-022-08458-9

**Published:** 2022-03-16

**Authors:** Randy Matos, Nezih Pala

**Affiliations:** FIU College of Engineering and Computing, 10555 W Flagler St, Miami, FL 33174 USA

**Keywords:** Engineering, Nanoscience and technology

## Abstract

As demand for higher capacity wireless communications increases, new approaches are needed to improve capacity. The lack of configurable radio platforms and power consumed to create new signals are some of the limitations preventing further advancements. To address these limitations, we propose an Ultra-Reconfigurable Intelligent Surface (URIS) platform based on the metal-to-insulator transition property of VO_2_. A VO_2_ layer is placed on a high-density micro-heater matrix consisting of pixels that can be electronically switched on. With this manner of control, heat can be transferred to selected areas of the VO_2_ layer and convert it to highly conductive metallic phase. This technique allows dynamically changing the shape of the reflection surface with high speed. We numerically investigated the heat activated switching and RF reflection characteristics of a reflectarray designed for potential 5G applications operating at 32 GHz. It consists of heating pixels with the size of 40 × 40 μm which can generate metallic VO_2_ patches or arbitrary shapes with ~ 100 × 100 μm spatial resolution. Our analyses resulted in large phase range of ~ 300° and approximate losses of −2 dB. The proposed device can serve as a novel platform for ultra-reconfigurable reflectarrays, other IRSs, and various wide spectral range RF applications.

## Introduction

Recent studies show that data traffic in communications technology is expected to quadruple from 2019 to 2025^1^. The general approach to address the demands for higher data rates in wireless communications is two-fold: (i) Moving to higher frequencies where larger bandwidths are available and (ii) increasing spectral efficiency by developing advanced multiplexing techniques. Although these approaches greatly benefited the emerging 5G technologies, they traditionally treated propagation medium between the transmitter and the receiver as a randomly behaving entity. However, recent developments in reconfigurable intelligent surfaces (RIS) make it possible to control the scattering and reflection of wireless signals and mitigate the negative effects of the propagation medium. RIS can effectively control the wavefront, by manipulating the phase, amplitude, frequency, and even polarization, of the impinging signals without the need of complex decoding, encoding, and radio frequency processing operations^2–8^. Implementation of RIS can also help reducing the power consumption by reusing the signals as opposed to creating new signals whenever data must be transmitted^9,10^.

Reconfigurable reflectarrays (RRAs), liquid crystal surfaces and software defined metasurfaces are among the implementation methods for RIS explored in the literature^2^. Despite the recent intense research, implementations that rely on electronic beam steering are still in their infancy. Mechanical beam steering has its uses^11^, but suffers from the arrays being designed for a specific polarization and the whole structure needs to be rotated or displaced in some manner. RRAs allow electronic beam steering by changing connections between the patch elements trough electronic signals in real time. Use of lumped components and tunable materials are the two major paradigms of current and emerging reconfigurable reflectarray antennas. The most common lumped components associated with developing RRAs are RF MEMS, PIN diodes, and varactor diodes^12–15^. Combinations of lumped components are also found in the literature^16^. For tunable materials, liquid crystals, ferroelectrics, and graphene have been proposed^17–20^. A summary of the properties of these recently proposed RRAs can be found in Table 1. The major hurdles in developing highly reconfigurable surfaces using tunable materials is to keep reflection losses as low as possible, reflection phase range as wide as possible, and being able to achieve wide beam widths such that all the patch elements can receive the incident signals from distant feeds^21^. Moreover, the implementation techniques reported in the literature are based on connecting/disconnecting predefined conducting patches by turning on/off the lumped elements and therefore have limited reconfigurability to only a handful of discrete states and/or within in a certain bandwidth. This is evident from the comparisons on Table 1, where the number of unit-cell states is either a fixed quantity, or a small variable range. Use of tunable materials such as Vanadium Dioxide (VO_2_) for RRAs can address these challenges. As a metal-to-insulator transition (MIT) material, VO_2_ transitions to a metallic phase when it is heated above a critical temperature of ~ 68 °C (342 K), though the transition begins around ~ 66.8 °C (340 K). In such phase transitions (switching), its electrical resistivity can be varied from 0.1 to 3 × 10^−6^ Ω m in a few nanoseconds. This transition occurs due to a change in its crystal structure, which changes from monoclinic to a tetragonal structure^22–25^. This temperature requirement is drastically different from current RF switches based on phase change materials (PCMs). Germanium antimony telluride (Ge_x_Sb_y_Te_z_), one of the commonly used PCMs, requires temperatures of ≈750 K^26^ to reach crystallization. Further heating is required to reach an amorphous state, ≈873 K^27^. We recently proposed an ultra-reconfigurable antenna platform based on a VO_2_ thin layer integrated with an electronically controlled resistive heating matrix^28^. To the best of our knowledge, VO_2_ has not been studied for highly reconfigurable intelligent surfaces in the literature. We believe that an ultra-reconfigurable intelligent surface reflectarray antenna could mitigate the limitations associated with the implementations reported in the literature, in particular the limited reconfigurability.

**Table 1:** Properties of recently proposed RRAs.

Reconfigurability method	Frequency range [GHz]	# of unit-cell states	Phase range	References
RF-MEMS	10.8–11.8, 14–15.4	Variable	300–360°	^12^
PIN diode	12.9–16.5	2	180 ± 20°	^13^
PIN diode	5.8	4	180°	^14^
Varactor	19.3–20.2	Variable	290–300°	^15^
PIN diode and varactors	7.5	2; Variable	285°	^16^
Liquid crystal	9.35	Variable	300°	^17^
Liquid crystal	23.8	Variable	150°	^18^
Ferroelectric	60	Variable	N/A	^19^
Graphene	275	4	180°	^20^

This paper proposes a novel, Ultra-Reconfigurable Intelligent Surface (URIS) in the form of a reflectarray based on the unique phase-change properties of VO_2_ and presents its detailed numerical study of thermal and RF characteristics. The proposed URIS can generate virtually any shape, size and distribution of conducting patches very much like generating an image on screen and therefore allows virtually unlimited states in a very large spectral band. We targeted the frequencies for 5G and beyond since it is the next milestone in wireless communications. Smart 5G environments are of crucial importance since electromagnetic waves struggle with blockage from objects that are significantly larger in size than the wavelength. Given that 5G is in the mmWave range, objects such as humans or furniture can present blockage. It has been shown that human blockage can penalize the link budget by up to 20–30 dB^29^. Hence, the motivation for the proposed URIS is that it can facilitate smarter wireless communication environments to address the growing need for bandwidth and energy efficiency, and to help mitigate the shortcomings of the current technology by tailoring the desired wavefronts through virtually unlimited reconfigurability. Further, the proposed URIS platform provides the capability of reconfiguring in real-time to fit the immediate needs presented by a dynamic environment and reducing effects of blockages in the emerging 5G applications.

The rest of the paper is organized as follows: The Theoretical Foundations section summarize the foundations that form the basis of the device which is followed by the Device Structure. Details of the method and results of the thermal analysis of the device are presented in the Thermal Analysis section. Following, the methodology for extracting the S-curve of the reflection phase and the results are presented in the Unit Cell Simulations section. Finally, conclusions are drawn in the last section.

## Theoretical foundations

Reflectarrays are designed using arrays of reflecting elements^30,31^. A reflectarray can be flat, or have a slight curve to it, with an illuminating feed antenna that illuminates the radiating elements. These elements consist of pre-designed patches, waveguides, rings, and dipoles that are used to reradiate the signal with more strength for a designed range^32–34^. The spatially designed elements are such that the incident field is scattered with an electrical phase that creates a planar phase front for a given far-field distance. There are different types of reflectarray types that use different design paradigms including dielectric arrays, metallic arrays, wave guide arrays and microstrip arrays^30,35–40^.

**Figure 1:**
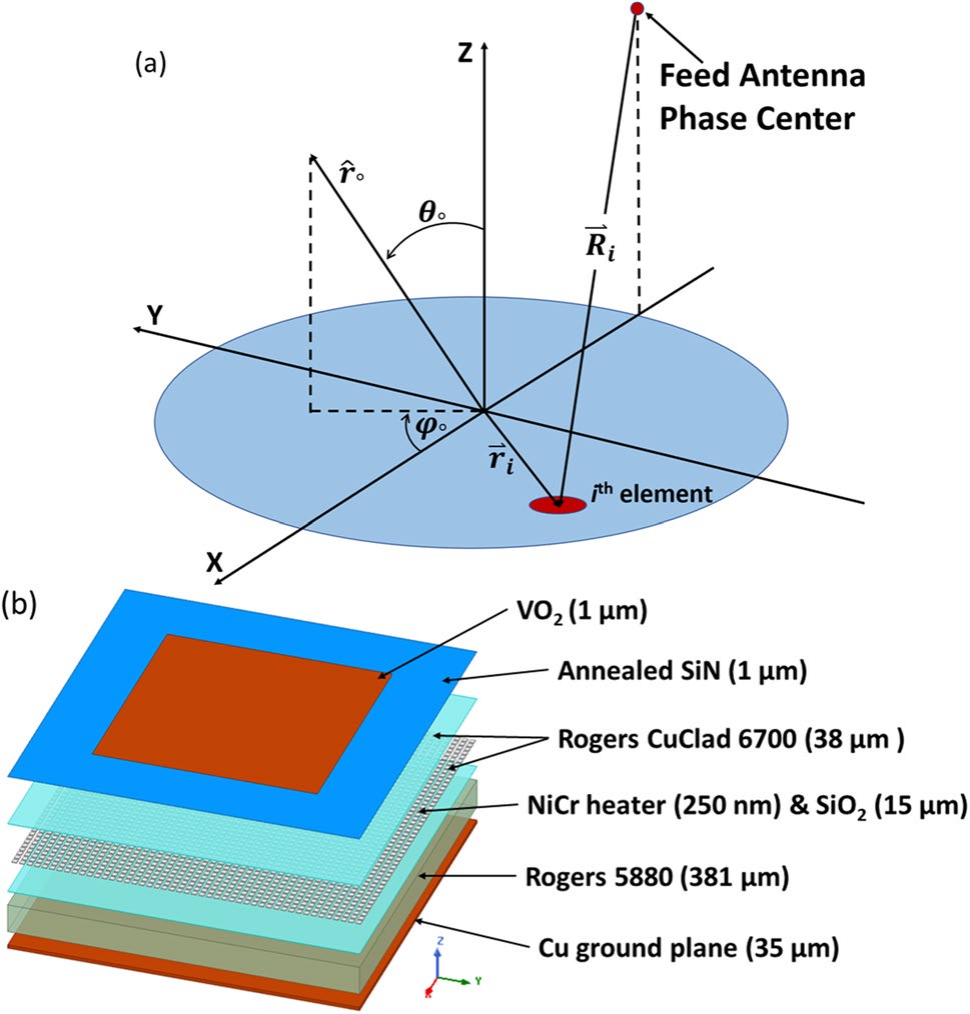
(**a**) Diagram describing differential spatial phase delay of a reflectarray. (**b**) Exploded device structure with materials.

Microstrip arrays combine the advantages of a dielectric and metallic reflectarray, which is to minimize conductor losses and to improve gain and efficiency, respectively^35,37,38^. Microstrip reflectarrays also have advantages in achieving low reflecting surface profiles, small antenna mass, and low manufacturing cost. Due to reflectarrays not requiring a power divider, they can also achieve efficiencies of > 50%, while also allowing for both mechanical and electronic beam steering^21^. Electronic beam scanning can also be used, which removes the requirements of the high-loss beamforming networks as well as the high-cost transmit/receive amplifiers, found in typical reflection-based antennas, are no longer needed^41,42^. Despite the many advantages, there is also a key disadvantage in that the reflectarrays bandwidth is limited by two major factors: narrow bandwidth on patch elements, and the differential spatial phase delay^43,44^. While the patch elements are inherently limited to a percent of the bandwidth, some techniques have been used to increase that, such as thick substrates and stacking multiple patches^45,46^. Differential spatial phase delay is another matter entirely. Best described by Fig. 1a, which shows that as the distance (R*i*) from the feed phase center to the *i*th element changes, the reflection phase of a reflectarray element must compensate for the spatial phase delay. This can be represented mathematically by:

1 $$\phi_{spd} = - k_{0} R_{i}$$

The wavenumber at the center frequency is represented by $k_{0}$. The spherical wave radiated by the feed is converted into a collimated beam in the Z direction by the given phase distribution. A progressive phase (pp) scan can be added to the aperture to scan the beam. In vector form, this can be represented as:

2 $$\phi_{pp} = - k_{0} \mathop{r_{i}}\limits^{\rightharpoonup} \cdot \hat{r}_{0}$$

where $\mathop{r_{i}} \limits^{\rightharpoonup}$ is the position vector of the *i*th element, and $\hat{r}_{0}$ is the direction of the main beam. This creates a frequency excursion error at the reradiated phase front, because the reflectarray design is created for phase compensation at the center frequency. The system presented in Fig. 1a can also be represented in a Cartesian system using (*x*_*i*_*,y*_*i*_), and for a beam represented in spherical coordinates as ($\theta_{0}$, $\varphi_{0}$), Eq. (2) simplifies to:

3 $$\phi_{pp} = - k_{0} \left( {x_{i} \sin \theta_{0} \cos \varphi_{0} + y_{i} \sin \theta_{0} \cos \varphi_{0} } \right).$$

The required phase shift, with the added progressive phase to compensate for the spatial phase delay, is given by:

4 $$\phi_{RA} = { }k_{0} \left( {R_{i} - \sin \theta_{0} \left( {x_{i} \cos \varphi_{0} + y_{i} \sin \varphi_{0} } \right)} \right) + \phi_{0} ,$$

where $\phi_{0}$ is a phase constant, expressing that, for reflectarray elements, a relative phase is needed. Some known solutions to reduce the amount of frequency excursion error exist, of which there are three main techniques to do so: design the array with a larger focal-length-to-diameter (*f*/*D*) ratio, avoid using a reflectarray with a large electrical diameter, and/or use time delay lines or partial time delay lines instead of the phase delays^21^. Most of these limitations stem from the need to have a fixed design.

## Device structure

Vanadium dioxide is a phase-change material (also referred as metal-to-insulator transition material) which behaves as an insulator at room temperature, but undergoes a phase transition to metallic state when heated above ~ 69 °C. To achieve this transition, various methods such as conductive heating, photo-thermal heating, Joule heating, and optical stimuli are used^22–25,47^. This transition has also been attained using static electric fields^48,49^. Due to the changes in resistivity and permittivity brought about by the transition, VO_2_ is an attractive material for applications in switching, optics, thermal diodes, antennas, waveguides, and resonators^23,28,47,50–54^.

In the investigated structure a VO_2_ layer is placed on a high-density micro-heater matrix consisting of pixels that can be switched on via electronic control. Controlling the pixels in this manner, we can transfer heat to the VO_2_ layer and heat up specific pixels to change the phase and hence the resistivity of the VO_2_^55^.

In contrast to the previous design reported in Ref.^28^, our initial studies showed that 6H-SiC could not be used directly as a substrate material due to its high dielectric constant of ε_r_ ~ 10.03, compared to conventional substrates like Rogers 5880 (ε_r_ = 2.2), Taconic TLX (ε_r_ = 2.4) or FR4 (ε_r_ = 4.4) as well as its high thermal conductance which limits the lateral patch resolution. To achieve high spatial resolution control and limit losses we developed and investigate the hybrid structure shown in Fig. 1b. The topmost layer consists of a uniform layer of VO_2_ (*t* = 1 μm). The second layer consists of Annealed SiN (*t* = 1 μm). The third layer is the heating matrix assembly which consists of a NiCr microheater (*t* = 250 nm) and Ni bias lines (*t* = 5 μm) embedded in SiO_2_ insulator layer (*t* = 15 μm). The final layer is Rogers 5880 laminate (ε_r_ = 2.2, *t* = 381 μm) with Cu back plating (*t* = 35 μm) to act as the RF ground plane. These layers, which cannot normally be fabricated together using conventional methods. will be bonded together using Rogers CuClad 6700 bonding film (*t* = 38 μm). The thermal behavior and RF performance of this device was analyzed numerically as explained in the following sections.

It should be noted that the proposed structure is not any more complex than other RRA implementations which require MEMS, lumped elements, or liquid crystals^12–20^. The only electrically active element of our design is the resistive heating matrix which is considerably simpler than the alternative implementations. Also, the proposed device structure can be fabricated using the current microfabrication techniques including photolithography, thin film deposition and wafer bonding. Operation of the proposed URIS would also be simple by a micro-controller, which would turn on and off the heating matrix elements to form the desired 2D surface geometry using pre-loaded patterns and algorithms.

## Thermal analysis

**Figure 2:**
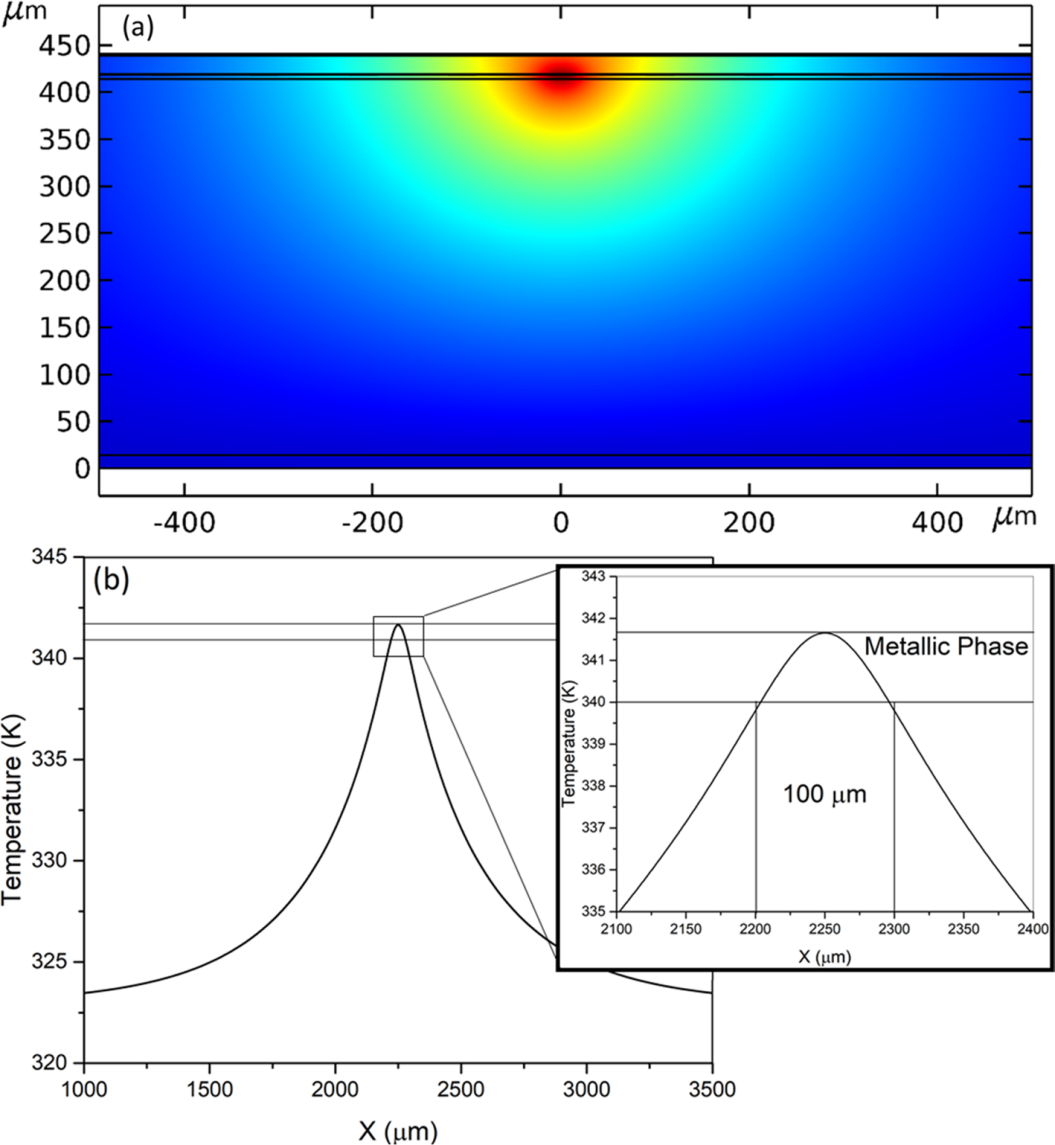
(**a**) 2D view of the thermal simulation of the device structure in Fig. 1b, which shows the thermal distribution of a single heater cell turned on. (**b**) Temperature curve of the top surface of the VO_2_ layer with a single heater on, with an inset showing the 100 μm resolution.

To verify thermal propagation of the device, as well as verify patch size, thermal simulations were performed using a commercial Multiphysics FEM tool (COMSOL™), its joule heating module with an electromagnetic heat source and heat transfer physics in solids. Figure 2 shows the thermal characteristics of the device in an “on” state after a steady state solution. This steady state for the heat transfer physics is described by:

5 $$\rho C_{p} u \cdot \nabla T = \nabla \cdot \left( {k\nabla T} \right) + Q$$

where *T*, *ρ*, *Cp*, *u*, *k* represents the temperature, mass density, heat capacity, velocity vector and thermal conductivity of the medium, respectively. This analysis allowed us to fine tune the distance between the micro-heaters such that the pixels’ transition edges just overlap enough to create a seamless and thermally homogeneous connection between pixels. Thermal analysis also allowed us to fine tune micro-heater parameters so that we could make it as efficient as possible. The unit cell dimensions within the simulation environment are 4.5 mm × 4.5 mm × 441 μm. Dimensions of the square heating element were set to 40 μm × 40 μm × 250 nm, of which there are 1681 heating elements (41 × 41). Because of the sheer number of elements, and the intensity of the simulation, only a 2D slice of the unit-cell is simulated. This approach is facilitated by the high symmetry across the device, and the software can take into account the depth of the device for calculation, while only numerically assessing the physics of the slice, therefore saving in computational resources and time, but maintaining accuracy in the simulation. This allows for a 2D slice that is spatially relevant, and would provide accurate thermal simulations of the device’s center and extremities. Both the top surface and rear surface of the device had convective losses and radiative losses considered. For convection boundaries, 10 W/m^2^K was selected as the heat transfer coefficient to represent a normal convection scenario, and the ambient temperature set to 293.15 ^o^K. For radiative boundaries, the emissivity of copper was selected as ε = 0.02. The emissivity of copper is a well-documented value and provided in the material parameters libraries. The emissivity of VO_2_ is chosen to be ε = 0.6 which is an experimentally measured value reported in the literature ^56^. It should be noted that the emissivity of VO_2_ changes with temperature, and that within this specific study, the emissivity change was ignored.

**Figure 3:**
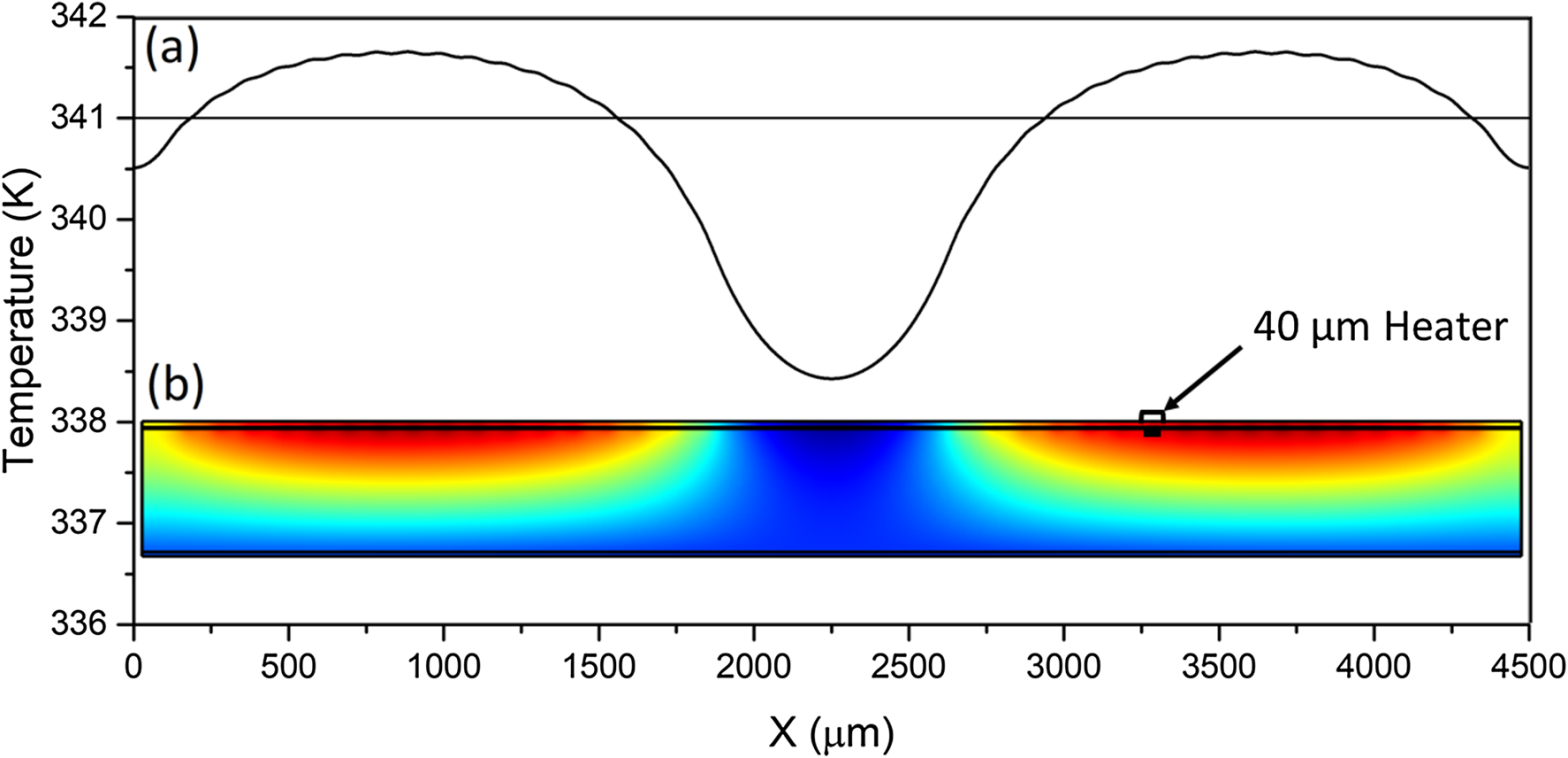
(**a**) Temperature curve of the top surface of the VO_2_ layer when multiple heaters are turned on to create two metallic patches. (**b**) 2D view of the simulation and thermal distribution during the same scenario. Size reference for the heater size is included.

Figure 2a shows the numerically calculated 2D thermal distribution where only a single heater cell is on. Surface temperature distribution for the same case is plotted in Fig. 2b. The results prove that it is possible to heat the top VO_2_ layer up to 341 ^o^K and thereby convert from insulating phase to highly conductive metallic phase, while staying under both the thermal limits of NiCr, and the thermal limits of the Rogers CuClad 6700 bonding layer. Figure 3a shows a 2D view of a two-patch scenario where 34, 40 μm-wide heaters are turned on and 7 are off to create two isolated, metal-transitioned patches of VO_2_ that are of 1200 μm width. This is at the lower end of patch size requirements for EM wave phase control at 32 GHz (see Fig. 5). Figure 3b shows the temperature spread across the surface during this two-patch scenario. The data shows that a gap of ~ 700 μm is needed to properly isolate patches due to reverse transition hysteresis. The total heat source power shown for the steady state solution is 17.3 μW for a potential of 19.5 mV. Which would correspond to a total heat source power of 30.5 mW for the unit cell of the reflectarray during a high-convection scenario. Using the same specifications, we performed comparative simulations to show that the power required to achieve the transition temperatures for Ge_x_Sb_y_Te_z_ is 352 μW for 751 K, and 591 μW for 878 K. This translates to 376 mW and 632 mW, respectively, for a unit cell of comparable size to the one presented in this manuscript.

**Figure 4:**
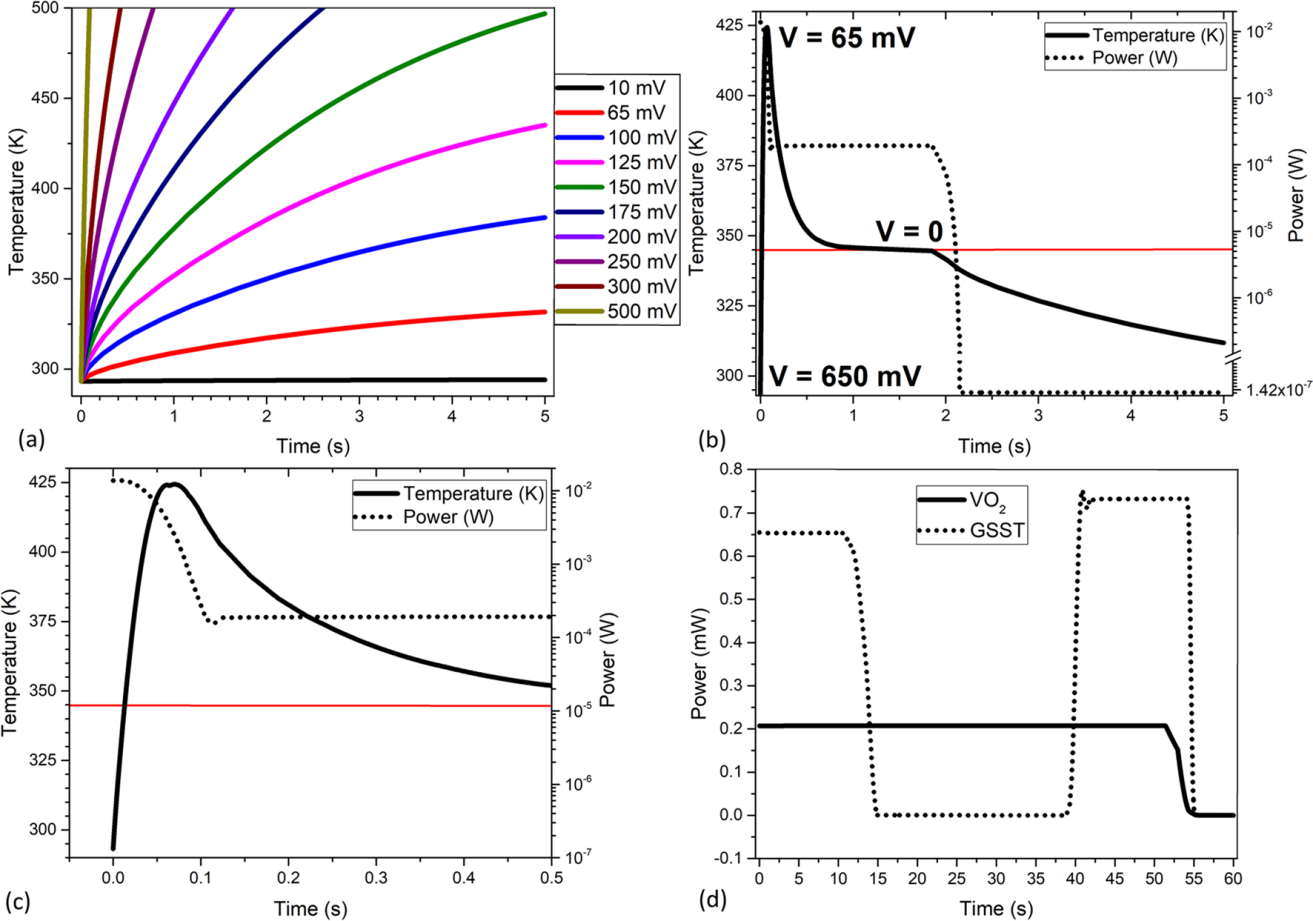
(**a**) Graph of the voltage dependence on the time to reach threshold temperature. (**b**) Temperature and power curves during numerical study applying 550 mV, stepping down to 65 mV, and removing the voltage. (**c**) Closer look into the temperature-power consumption curves from 0 to 500 ms. (**d**) Power draw comparisons for similar structures with comparable performance.

A time-dependent study was also conducted on the same structure. The time-dependent solution is given by:

6 $$\rho C_{p} \frac{\partial T}{{\partial t}} + \rho C_{p} u \cdot \nabla T = \nabla \cdot \left( {k\nabla T} \right) + Q,$$

where *T*, *t*, *ρ*, *Cp*, *u*, *k* represent the temperature, time, mass density, heat capacity, velocity vector and thermal conductivity of the medium, respectively. Figure 4a shows the ramp up time and temperature of the top VO_2_ surface for a range of voltages. The time-dependent analysis shows that the time to reach the threshold temperature is also configurable and can vary depending on design and power constraints. Figure 4b shows a numerical study where 550 mV was used to pass the threshold temperature within 12 ms and then stabilize at 343 ^o^K by stepping down the voltage to 65 mV in a high convection scenario where convection losses are 200 W/m^2^K. The data shows that the transition temperature can be reached very quickly, but that reaching the hysteresis reversion temperature takes longer at approximately 1 to 2 s. Even though that time is not very long, given design constraints, the cool down time can be improved by adding an active cooling layer to the bottom. Figure 4c shows the enlarged section of 0 to 500 ms at Fig. 4b.

A time-dependent study was also conducted where a similar structure of Ge_x_Sb_y_Te_z,_ was brought up to the required temperature to crystallize it and then brought up to amorphization temperature within 60 s. The power draw was then compared to that of the proposed structure using VO_2_. The results of that study are shown in Fig. 4d. This comparison is important since Ge_x_Sb_y_Te_z_ can crystallize and maintain its properties with no further power draw means that for very long, extended periods of time, Ge_x_Sb_y_Te_z_ may have the power advantage as it would not need the constant, albeit very low, power draw of VO_2_ to maintain its conductive state. However, the data shows that VO_2_ may have the advantage in high switching scenarios due to its much lower power draw. These results show that we can achieve fast transition times, which are needed for real-time reconfiguring of the patch size, shape, and distribution, while maintaining competitive power requirements.

## Unit-cell simulation

**Figure 5:**
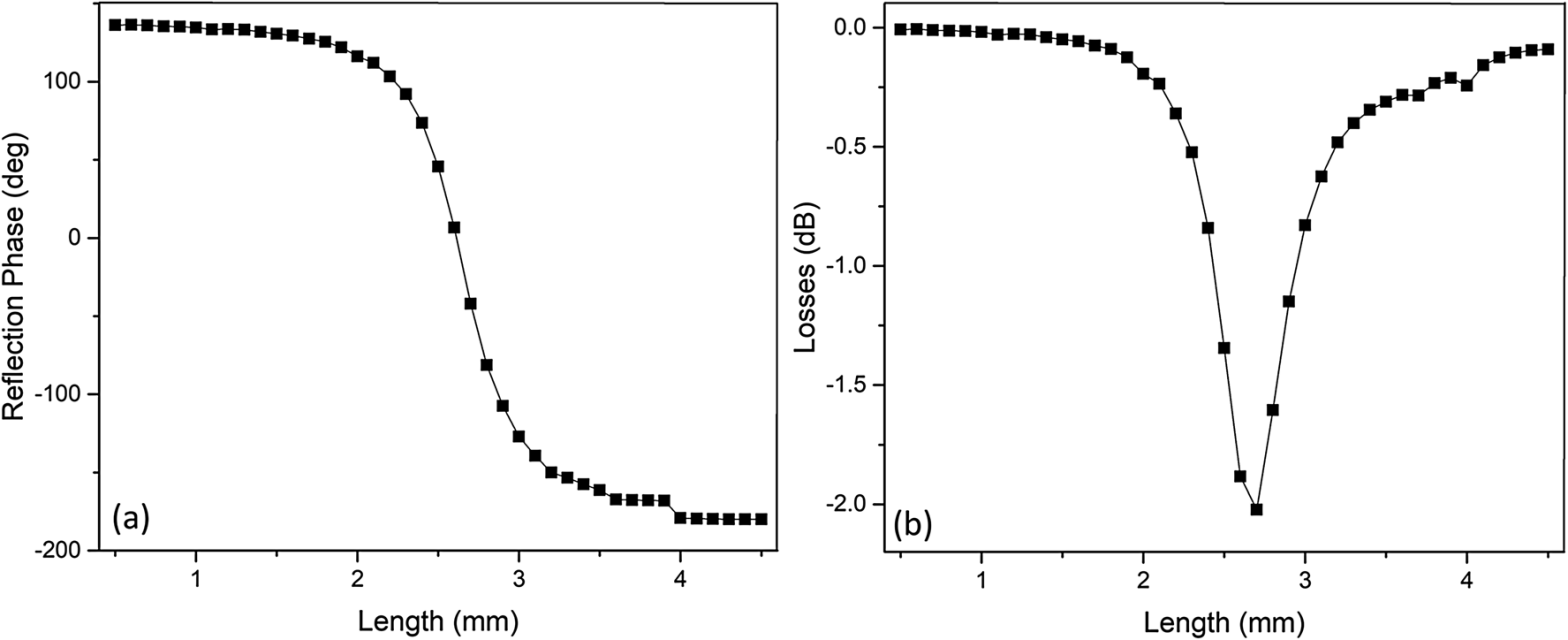
Reflection phase curve (**a**) and loss curve (**b**) of the simulated hybrid structure for 32 GHz.

We investigated the RF characteristics of the proposed device for 32 GHz and 5 GHz which are part of the frequency bands for 5G applications. A square geometry for the reflective surface is selected since the square geometry for reflectarray unit-cells is highly characterized and well-studied. It should be noted that our device platform is capable of creating almost any 2D pattern (i.e. circular, multi-ring, cross, dipole, etc.). The most efficient way to design a reflectarray antenna is to begin with a unit cell analysis of the reflectarray. The unit cell is the fundamental unit that makes the whole of the array. A phase design curve can be obtained by changing the physical characteristics of the unit cell and monitoring its phase response. We used Ansys HFSS for the numerical analysis of the RF reflection characteristics of the proposed unit cell. The master and slave boundaries are used as the periodic boundary conditions, and the excitation ports are implemented using Floquet ports. To verify our simulation method, reported characteristics of the design described in Ref.^55^ for 32 GHz operation using conventional RF materials were replicated. The inter-element spacing was selected as 0.5λ, where λ is the free-space wavelength at 32 GHz. The element consists of a square VO_2_ patch that varies in length and width from 0.5 mm to 4.5 mm at an interval of 0.1 mm. We assumed the VO_2_ layer resistivity was ρ = 10^–5^ Ω.m, which is a conservative value compared to the experimentally achieved ones^58,59^. Figure 5a shows the reflection angle as a function of the square patch size of the investigated structures and its comparison with the reference design. The characteristics show a well-defined S-curve typical for reflection arrays covering a large phase range of ~ 300°. Figure 5b shows the losses for the same analysis. The obtained reflection losses of ~ -2 dB are considered very high efficiency within the context of reflectarrays^60^.

**Figure 6:**
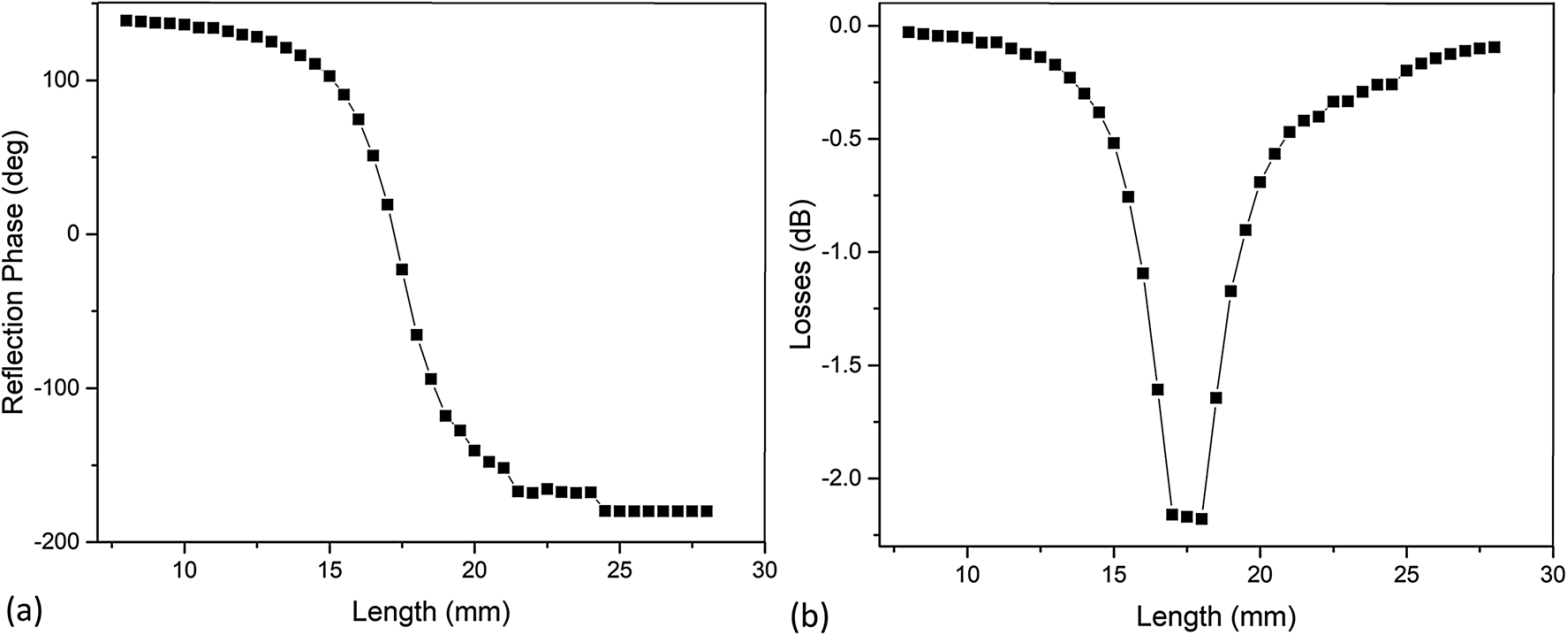
Reflection phase curve (**a**) and loss curve (**b**) of the simulated mid-band hybrid structure for 5 GHz.

We also probed mid-band 5G range which is current communications technology. This range is considered between 1 and 6 GHz. We designed a reflectarray unit cell using our VO_2_-based URIS and simulated its reflection phase S-curve. Due to the change in frequency, the dimensions of the reflectarray patch would have to change. The inter-element spacing was selected as 0.5λ, where λ is the free-space wavelength at 5 GHz. The element consists of a square patch that varies in length and width from 8 to 28 mm at an interval of 0.5 mm. The thickness of the base Rogers 5880 layer had to also become thicker to accommodate for the lower frequency. The new thickness was 3 mm of Rogers 5880. All other layers stayed the same. Figure 6a shows the reflection phase curves, and Fig. 6b shows the reflection loss curves. The characteristics show a well-defined S-curve, like with the previous design, covering a large phase range of 320°, and a reflection loss −2.18 dB. The results show that the proposed platform also performs well at lower frequencies for immediate wireless applications.

## Conclusion

We proposed an ultra-reconfigurable intelligent surface platform based on the phase-change properties of VO_2_ and presented detailed study of thermal and RF characteristics of its unit cell. The investigated designs presented smooth reflection phase control curve covering ~ 300° and reflection loss no greater than −2 dB. The proposed platform allows generating conducting patches in any size, shape, and distribution, very much like generating an image on a screen, within the timescale of milliseconds. The results show the feasibility of our novel VO_2_ based, heating matrix integrated ultra-reconfigurable reflectarray as a promising platform for reconfigurable intelligent surface applications for both mid-band and high band 5G spectra.

## Data Availability

The datasets generated during and/or analyzed during the current study are available from the corresponding author on reasonable request.
